# Resting Heart Rate and Risk of Cardiovascular Diseases and All-Cause Death: The Kailuan Study

**DOI:** 10.1371/journal.pone.0110985

**Published:** 2014-10-24

**Authors:** Anxin Wang, Shuohua Chen, Chunxue Wang, Yong Zhou, Yuntao Wu, Aijun Xing, Yanxia Luo, Zhe Huang, Xiaoxue Liu, Xiuhua Guo, Xingquan Zhao, Shouling Wu

**Affiliations:** 1 Department of Neurology, Beijing Tiantan Hospital, Capital Medical University, Beijing, China; 2 Department of Epidemiology and Health Statistics, School of Public Health, Capital Medical University, Beijing, China; 3 Beijing Municipal Key Laboratory of Clinical Epidemiology, Capital Medical University, Beijing, China; 4 Department of Cardiology, Kailuan Hospital, Hebei United University, Tangshan, China; 5 Department of Cardiology, Tangshan people’s Hospital, Tangshan, China; Indiana University School of Medicine, United States of America

## Abstract

**Background:**

Resting heart rate (RHR) predicts both cardiovascular and noncardiovascular death in different populations. However, the results of the association between RHR and cardiovascular diseases (CVDs) are inconsistent, especially for each subtype of CVDs.

**Objective:**

The aim of this study was to prospectively explore the relationship between RHR and CVDs including myocardial infarction (MI), ischemic stroke, and hemorrhagic stroke and all-cause death in a general population.

**Methods:**

The Kailuan study is a prospective longitudinal cohort study on cardiovascular risk factors and cardiovascular or cerebrovascular events. Hazard ratio (HR) with 95% confidence intervals (CI) were calculated using Cox regression modeling.

**Results:**

We analyzed 92,562 participants (18–98 years old) in the Kailuan Study. CVDs were developed in 1,903 people during follow-ups. In multivariate analysis with adjustment for major traditional cardiovascular risk factors, HRs of the highest quintile group compared with the lowest quintile group of RHR for all-cause CVDs, MI, any stroke, ischemic stroke, hemorrhagic stroke, and all-cause death were 1.03 (95% CI, 0.98–1.07), 1.10 (95% CI, 1.01–1.20), 1.01 (95% CI, 0.97–1.06), 1.02 (95% CI, 0.96–1.07), 1.01 (95% CI, 0.92–1.11) and 1.18, (95% CI, 1.13–1.23), respectively.

**Conclusions:**

The elevated RHR was independently associated with the increased risk for MI and all-cause death, but not for all-cause CVDs, any stroke, ischemic stroke, nor hemorrhagic stroke. This indicates that the elevated RHR might be a risk marker for MI and all-cause death in general populations.

## Introduction

A number of epidemiological and clinical studies suggest that resting heart rate (RHR), a simple and easily measurable clinical parameter, predicts both cardiovascular and noncardiovascular death in general populations [1], [2], [3], [4], [5], [6], [7], [8], [9], and in patients with coronary artery disease [10], [11], stroke [12], [13], and hypertension [14], [15]. However, the results of the association between RHR and cardiovascular diseases (CVDs) are inconsistent. Some previous studies indicated that a high RHR was an independent risk factor for all-cause CVDs [10], [16], [17], myocardial infarction (MI) [10], [12], and stroke (including ischemic stroke and hemorrhagic stroke) [10], [12], [16] after adjusting the most major cardiovascular risk factors. Other studies have not detected the significant relationship between the elevated RHR and the risk of all-cause CVDs [18], MI [1], and stroke [1].

Thus, in this study, we prospectively examined the relationship between RHR and the risk of CVDs including MI, ischemic stroke, and hemorrhagic stroke in the Kailuan Study.

## Methods

### Study design and population

The Kailuan Study is a prospective population-based cohort study involving 101,510 men and women aged 18–98 years in the Kailuan community in Tangshan of China. The design and methods of the study have been described in detail previously [19], [20], [21]. The protocol was approved by the Ethics Committee of both Kailuan General Hospital and Beijing Tiantan Hospital in compliance with the Declaration of Helsinki.

In brief, all participants provided a written informed consent and underwent a clinical examination and a standardized interview including questions on smoking and alcohol consumption status, socioeconomic parameters, physical activity, histories of arterial hypertension, coronary heart disease, diabetes mellitus, hyperlipidemia, and stroke, with the current treatments of these diseases. Anthropomorphic parameters, such as body height, weight, and waist circumference were measured. The body mass index was calculated as the ratio of body weight (kilograms) divided by the square of body height (meters). Blood pressure was measured on the left arm to the nearest 2 mm Hg using a mercury sphygmomanometer with a cuff of appropriate size. Fasting blood samples were biochemically examined for the concentration of glucose, high-density lipoproteins (HDL), low-density lipoproteins (LDL), triglycerides, total cholesterol, and high-sensitive C-reactive protein (hs-CRP).

Hypertension was defined as having a history of hypertension, a systolic blood pressure (SBP) ≥140 mmHg, a diastolic blood pressure (DPB) ≥90 mmHg, or using antihypertensive medications. Diabetes mellitus was diagnosed if the subject had a history of diabetes mellitus, was currently using insulin or oral hypoglycemic agents, or had a fasting blood glucose level was ≥126 mg/dL. Hyperlipidemia was defined as having a history of hyperlipidemia, total blood cholesterol levels ≥220 mg/dL, triglyceride levels ≥150 mg/dL, or taking antihyperlipidemic medications.

The study participants were refrained from smoking and drinking of coffee, tea, or alcohol for at least three hours nor performing any exercise for at least 30 minutes prior to the RHR measurement. A 10-second 12-lead electrocardiography was used to measure the RHR after the participants rested in supine position for 5 minutes. The number of R-R intervals (number of QRS complexes - 1) was divided by the time difference between the first and last beat, and the result was converted to beats per minute (bpm) [20].

The interview and all examinations were carried out by specially trained medical doctors and nurses.

### Follow-ups and outcomes

The participants were followed up by face-to-face interviews at every two-year medical examination routine until December 31, 2010 unless CVDs events or death occurred. The follow-ups were performed by hospital physicians, research physicians, and research nurses. For the participants without face-to-face follow-ups, their outcome information was obtained by referring to death certificates from provincial vital statistics offices, discharge summaries from the 11 hospitals, or medical records from medical insurance companies [22], [23].

The primary outcome was the first occurrence of any CVDs including either the first nonfatal CVDs event or a CVDs death. Any stroke event included ischemic stroke or hemorrhagic stroke event The diagnosis of all CVDs events was confirmed through a medical record review, using the World Health Organization criteria [24], [25].

### Statistical analyses

We stratified the study population into quintiles based on the RHR (Q1 group: participants with RHR ≤66 bpm, Q2: participants with the RHR range of 67–70 bpm, Q3 group: participants with the RHR range of 71–74 bpm, Q4: participants with the RHR range of 75–80 bpm, and Q5 group: participants with RHR ≥81 bpm). Continuous variables were described by means ± standard derivation (SD) and categorical variables were described by percentages. We used the ANOVA test for non-paired samples of normally distributed parameters and the Kraskal-Wallis test for non-parametric variables. The Chi-squared test was applied for the comparison of categorical variables. We used a Cox proportional hazards model to calculate hazard ratios (HRs) and their 95% confidence intervals (CIs) for all CVD, stroke, MI, ischemic stroke, hemorrhagic stroke and all-cause death, respectively by treating the lowest RHR quintile (Q1 group) as the reference category. Person-years of follow-up were calculated as the time from baseline assessment to either the development of the first endpoint of interest, censoring, or the end of follow-ups (December 31, 2010). We created three multivariate-adjusted models. Model 1 was adjusted for age and sex. In addition to adjusting for the confounders in Model 1, Model 2 also adjusted for the following confounders: average monthly income of each family member, education level, marital status, body mass index, waist circumference, smoking status, drinking status, and physical activity., Model 3 adjusted for hs-CRP, hypertension, diabetes mellitus, and dyslipidemia apart from the confounders that were adjusted in Model 2.

Finally, we also conducted the above three Models by treating the RHR as a continuous variable in order to test the liner relationship between RHR and CVD events.

Two-sided *P*-values were reported for all analyses. A *P*-value <0.05 was considered to be statistically significant. All statistical analyses were performed by SAS software, version 9.3 (SAS Institute Inc., Cary, NC, USA).

## Results

Out of the 101,510 participants who were originally included in the Kailuan study, we excluded 3,669 participants with a prior history of MI or stroke, and 5,279 without relative data at baseline. Therefore, 92,562 participants including 73,938 men and 18,624 women were in this analysis.

During a mean follow-up of four years, 1,903 participants suffered from CVDs and 1,589 had all-cause death. Among the participants with CVDs, 399 had MI, 1,519 had stroke including 1,122 ischemic stroke events and 397 hemorrhagic stroke events. Baseline characteristics of the entire study cohort stratified by the RHR quintiles are presented in Table 1. Statistically significant differences among the RHR quintiles were found for the following variables: age, gender, body mass index, waist circumference, marital status, education level, average income of each family member, physical activity, smoking status, drinking status, prevalence of hypertension, diabetes mellitus and dyslipidemia, and the level of hs-CRP.

**Table 1:** Participants baseline characteristics according to quintiles of resting heart rate.

	Quintiles of resting heart rate					
	Q1	Q2	Q3	Q4	Q5	p
	< = 66 bpm	67–70 bpm	71–74 bpm	75–80 bpm	> = 81 bpm	
No. ofparticipants	19060	19574	14159	23506	16263	
Male, %	80.59	79.30	76.70	80.46	81.66	<0.0001
Mean age(years) (SD)	52.45(12.57)	50.31(12.18)	51.72(12.22)	50.47(12.34)	51.09(12.95)	<0.0001
Mean body massindex (kg/m^2^ )(SD)	24.75(3.35)	25.10(3.40)	25.11(3.52)	25.14(3.52)	24.99(3.68)	<0.0001
Mean waistcircumference(cm) (SD)	86.62(10.10)	86.68(9.67)	86.94(10.22)	87.38(10.02)	87.33(10.46)	<0.0001
Marital status(married) (%)	94.76	95.05	94.36	94.92	94.26	0.0022
Over or equal tohigh-schooleducation (%)	20.95	19.10	20.72	18.89	19.38	<0.0001
Average incomeof each familymember ≥800Y– (%)	15.67	12.89	14.46	12.56	13.87	<0.0001
Physical activity>4 times/week (%)	15.42	14.49	17.97	14.41	11.28	<0.0001
Past/currentsmoker (%)	39.29	37.52	38.79	39.39	40.26	<0.0001
Past/currentalcohol drinker(%)	42.80	38.56	40.08	39.87	40.17	<0.0001
Hypertension(%)	34.80	41.30	41.37	45.82	50.76	<0.0001
Diabetes mellitus(%)	5.83	7.38	8.48	9.55	13.96	<0.0001
Dyslipidemia(%)	30.21	34.32	33.46	36.58	38.90	<0.0001
Medianhigh-sensitiveC-reactive protein(IQR) (mg/dl)	0.71(0.30–20.00)	0.80(0.30–2.28)	0.81(0.30–2.30)	0.80(0.30–2.00)	0.90(0.33–2.50)	<0.0001

bpm, beats per minute; SD, standard deviation; IQR, inter quartile range.

### All-cause cardiovascular disease

Compared with the lowest RHR group, The highest HR (95% CI) for the risk of all-cause CVDs was 1.40 (1.21–1.61) in Model 1 and 1.39 (1.20–1.61) in Model 2, respectively (Table 2). However, there was no significant relationship between RHR and all-cause CVD after adjusting all confounders in Model 3. After treating heart rate as a continuous variable, the results were similar.

**Table 2:** Hazard ratios (95% CI) for cardiovascular disease and all-cause death according to quintiles of resting heart rate.

	Quintiles of resting heart rate					Restingheart rateper 10 bpm
	Q1	Q2	Q3	Q4	Q5	
	< = 66 bpm	67–70 bpm	71–74 bpm	75–80 bpm	> = 81 bpm	
No. of subjects	19060	19574	14159	23506	16263	
All-cause cardiovascular diseases						
Cases (%)	349(1.83)	372(1.90)	303(2.14)	491(2.09)	388(2.39)	
Model 1†	reference	1.21(1.05–1.41)	1.26(1.08–1.47)	1.30(1.14–1.50)	1.40(1.21–1.61)	1.10(1.06–1.14)
Model 2‡	reference	1.20(1.03–1.39)	1.25(1.07–1.46)	1.26(1.10–1.45)	1.39(1.20–1.61)	1.10(1.05–1.14)
Model 3§	reference	1.12(0.96–1.29)	1.15(0.98–1.35)	1.14(0.99–1.31)	1.13(0.97–1.31)	1.03(0.98–1.07)
Myocardial infarction						
Cases (%)	70(0.37)	71(0.36)	62(0.44)	99(0.42)	97(0.60)	
Model 1†	reference	1.19(0.86–1.66)	1.31(0.93–1.84)	1.34(0.99–1.82)	1.74(1.28–2.36)	1.15(1.06–1.25)
Model 2‡	reference	1.22(0.87–1.71)	1.34(0.94–1.89)	1.34(0.98–1.83)	1.80(1.31–2.46)	1.16(1.06–1.26)
Model 3§	reference	1.14(0.81–1.59)	1.22(0.86–1.74)	1.21(0.88–1.66)	1.45(1.05–1.99)	1.10(1.01–1.20)
Any stroke						
Cases (%)	281(1.47)	302(1.54)	245(1.73)	396(1.68)	295(1.81)	
Model 1†	reference	1.21(1.03–1.43)	1.26(1.06–1.49)	1.30(1.11–1.51)	1.31(1.11–1.55)	1.09(1.04–1.14)
Model 2‡	reference	1.18(1.00–1.40)	1.24(1.04–1.47)	1.24(1.06–1.45)	1.29(1.10–1.53)	1.09(1.04–1.14)
Model 3§	reference	1.10(0.93–1.30)	1.14(0.96–1.36)	1.12(0.96–1.31)	1.05(0.89–1.24)	1.01(0.97–1.06)
Ischemic stroke						
Cases (%)	205(1.08)	215(1.10)	190(1.34)	297(1.26)	215(1.32)	
Model 1†	reference	1.20(0.99–1.45)	1.35(1.10–1.64)	1.35(1.13–1.61)	1.32(1.09–1.59)	1.10(1.04–1.16)
Model 2‡	reference	1.17(0.97–1.43)	1.33(1.09–1.62)	1.30(1.08–1.56)	1.31(1.08–1.59)	1.10(1.04–1.16)
Model 3§	reference	1.08(0.89–1.31)	1.21(0.99–1.48)	1.16(0.96–1.39)	1.03(0.85–1.26)	1.02(0.96–1.07)
Hemorrhagic stroke						
Cases (%)	76(0.40)	87(0.44)	55(0.39)	99(0.42)	80(0.49)	
Model 1†	reference	1.24(0.91–1.69)	1.02(0.72–1.44)	1.16(0.86–1.56)	1.30(0.95–1.78)	1.06(0.97–1.16)
Model 2‡	reference	1.21(0.89–1.65)	1.00(0.70–1.41)	1.09(0.80–1.48)	1.24(0.91–1.71)	1.05(0.96–1.15)
Model 3§	reference	1.15(0.84–1.58)	0.95(0.67–1.35)	1.02(0.75–1.39)	1.11(0.80–1.53)	1.01(0.92–1.11)
All-cause death						
Cases (%)	276(1.45)	270(1.38)	232(1.64)	436(1.85)	375(2.31)	
Model 1†	reference	1.16(0.98–1.37)	1.24(1.04–1.48)	1.51(1.30–1.76)	1.74(1.49–2.03)	1.23(1.18–1.28)
Model 2‡	reference	1.15(0.97–1.36)	1.23(1.03–1.47)	1.49(1.28–1.73)	1.70(1.45–1.99)	1.22(1.17–1.27)
Model 3§	reference	1.09(0.92–1.29)	1.18(0.99–1.41)	1.38(1.19–1.61)	1.51(1.29–1.77)	1.18(1.13–1.23)

CI, confidence interval; bpm, beats per minute.

† Model 1 adjusted for age and sex.

‡ Model 2 adjusted for age, sex, average monthly income of each family member, education level, marital status, body mass index, waist circumference, smoking status, drinking status, and physical activity.

§ Model 3 adjusted for age, sex, average monthly income of each family member, education level, marital status, body mass index, waist circumference, smoking status, drinking status, physical activity, high-sensitive C-reactive protein, hypertension, diabetes mellitus, and dyslipidemia.

### Myocardial infarction

After controlling for all the potential confounders in Model 3, participants in the highest RHR group (≥81 bpm) had a statistically significant increased risk of 45% for MI when compared with those in the lowest RHR group (≤66 bpm) (Table 2). The risk of MI was significantly increased by 10% with an increase of 10 bpm (Table 2).

### Any stroke, ischemic stroke, and hemorrhagic stroke

There was a significant association between increased RHR and any stroke and ischemic stroke in Model 1 and Model 2. But after adjusting for all confounders in Model 3, the relationship disappeared completely (Table 2). There was no significant association between RHR and hemorrhagic stroke in the three Models (Table 2).

### All-cause death

All-cause mortality was increased in a graded trend from the lowest to the highest RHR group (1.45%, 1.38%, 1.64%, 1.85%, and 2.31%, respectively) (Table 2). After adjusting for all confounders, participants in the Q4 group (75–80 bpm) and those in the Q5 group (≥81 bpm) had more risk for all-cause death than those in the reference group (≤66 bpm) and HRs were 1.38 (95% CI, 1.19–1.61) and 1.51 (95% CI, 1.29–1.77), respectively. After treating heart rate as a continuous variable, the risk for all-cause death was increased by 18% per 10 bpm (HR: 1.18 [95% CI, 1.13–1.23]) after adjusting all confounders (Table 2).

## Discussion

In this large prospective cohort study of 92,526 participants, we found that RHR was independently associated with the risk of MI and all-cause death after controlling for potential confounders such as age, sex, body mass index, waist circumference, smoking status, drinking status, physical activity, hs-CRP, hypertension, diabetes mellitus, and dyslipidemia. However, there was no significant association between RHR and the risk of all-cause CVDs, any stroke, ischemic stroke, and hemorrhagic stroke after adjusting all confounders.

Some previous studies suggested that there was a significant relationship between RHR and all-cause CVDs [10], [16], [17]. However, some other studies found an elevated RHR was not associated with the increased risk of all-cause CVDs after adjustment for potential cardiovascular risk factors [18], which is consistent with our findings. This may indicate that RHR is not an independent risk factor for all-cause CVDs.

We found that the elevated RHR was significantly associated with increased risk of MI in our study. This result was similar with some previous studies [10], [12].

Our study showed that there was no relationship between RHR and any stroke, ischemic stroke, or hemorrhagic stroke after controlling all potential risk factors. The results of many previous studies about this relationship were contradictory [1], [10], [12], [16].

The association between RHR and all-cause mortality was described in previous studies in different populations [1], [2], [3], [4], [5], [6], [7], [8], [9], [10], [11], [12], [13], [14], [15] and was consistent with our results. In spite of the existent large body of knowledge in this field, our present study included more potential confounders in the adjusted models and it could be further confirmed that RHR was an independent risk factor for all-cause death.

This study has several strengths, including the prospective design, large sample size in an Asian population, enrollment of women and men, broad assessment of potential confounders, and confirmation of CVDs events through reviewing medical records. However, potential limitations of our study should be noticed. First, the participants in the Kailuan study do not constitute a nationally representative sample and our findings may not be generalized directly to other Chinese populations with different educational and cultural backgrounds. Second, RHR was calculated from one electrocardiography record. It has been well known that RHR depends on the daytime. Assessment of the mean RHR obtained in several measurements during a 24 hour profile might have been resulted in data independent of the daytime.

## Conclusions

To summarize, we found an independent relationship between the elevated RHR and the risk of MI and all-cause death, which was attenuated after adjustment for major cardiovascular risk factors, but not for all-cause CVDs, any stroke, ischemic stroke, and hemorrhagic stroke. This indicates that the elevated RHR could be regarded as a risk marker for MI and all-cause death in general populations.
